# Supplementary material for: Endogenous Oxytocin, Vasopressin, and Aggression in Domestic Dogs
Source: Front Psychol. 2017 Sep 27;8:1613. doi: 10.3389/fpsyg.2017.01613 (PMC5624304; doi:10.3389/fpsyg.2017.01613)
Supplement: Supplementary file 1 [file DataSheet1.PDF]

Supplemental Information

Movie S1. Example of the procedure for Experiment 1. Web version available at:

https://www.youtube.com/watch?v=TlLrF2FbkOg&feature=youtu.be

Methodological Studies

Oxytocin samples were assayed using a commercially available enzyme-linked immunosorbent assay (ELISA) kits from Arbor Assays (K048) and Cayman Chemical (500440). Vasopressin samples were assayed using a commercially available ELISA kit from Enzo Life Sciences (ADI-900-017A).

Sample Extraction (Protocols for Experiment 1)

We first sought to determine whether sample extraction was required for the accurate measurement of free OT and AVP in dog plasma. To do so, we tested for parallelism using dog plasma samples with and without solid phase extraction (SPE).

Extraction Protocols.

Extracted samples were processed with Oasis PRiME HLB reversed-phase cartridges (Waters Corporation). Initially we followed the protocol developed by Cool and Debrosse (Cool & DeBrosse, 2003), with the modification that AVP fractions were eluted with 90% (as opposed to 80%) acetonitrile (ACN). This change was implemented following pilot studies with OT- and AVP-spiked buffer in which AVP was better recovered using 90% ACN (85% recovery as opposed to 60% recovery with 80% ACN). Recovery of OT using this protocol was ~100% with no detectable AVP in the OT fraction.

Linearity and Parallelism.

To test for linearity and parallelism we diluted pooled samples from 100% to 10% of their fully concentrated value in 10% increments. Due to the relatively low peptide concentrations in extracted samples, we concentrated samples during the extraction phase by running 1.5mL of plasma on each cartridge, but reconstituting these samples in only 260 µl assay buffer yielding

~6x concentration. Non-extracted samples were diluted beginning with neat plasma for OT and 1:4 plasma for AVP because preliminary tests revealed strong deviations from linearity for AVP samples assayed at concentrations > 1:2. Linearity was assessed by fitting a linear model to the observed values as a function of the expected values at each dilution. We expected that regressions of the observed concentration against the expected concentration would have slopes ~1, with an intercept that did not differ significantly from 0. The results of these analyses are shown in Table S1. Although the dilution factor accounted for a large proportion of variance in all cases (> 0.85), slopes for the non-extracted samples were higher than expected with intercepts differing significantly from 0, whereas slopes for extracted samples were close to 1 with intercepts that did not differ from 0.

Table S1. Slopes (β) for a series of diluted samples with and without sample extraction. The t and p values correspond to tests of the intercept against the predicted value of 0.

Peptide	Treament	β	Intercept	t	p
Oxytocin	non-extracted	1.55	-70.90	-4.52	**< .001**
	extracted	1.05	3.59	.71	.50
Vasopressin	non-extracted	1.59	-11.50	-2.31	**.05**
	extracted	.91	.60	.31	.76

Parallelism was assessed by plotting the logit of the percent binding against the expected sample value for dilutions of both the samples and standards. For both peptides the slopes for dilutions of extracted samples approximated those for the standards more so than slopes from dilutions of non-extracted samples. Based on these parallelism studies we opted to extract all samples for our analyses in Experiment 1.

Sample Extraction (Protocols for Experiment 2)

Prior to the main analysis of free OT and AVP in Experiment 2, we determined that the sequential elution procedure described above led to poorer recovery of AVP in dog plasma than when AVP was extracted without prior elution of the OT fraction. Therefore, we validated

additional protocols for extracting OT and AVP separately (on different columns). These methods were identical to those described above except that columns were conditioned with 1 mL ACN, and OT was eluted with 95% ACN / 5% H_20 (0.1% Trifluoroacetic acetic acid). In addition to this change in the SPE protocol, analysis of free OT (for the within assistance dog comparison) in Experiment 2 was performed with the Cayman Chemical OT kit, which permitted detection in a better range of the kit's standard curve. Below we report the results of validation studies with these modified procedures.

Linearity and Parallelism

As above, pooled plasma samples were assayed at multiple dilutions with and without extraction. We assessed linearity by fitting a linear model predicting the observed concentrations as a function of the expected concentrations at each dilution. The results from these analyses are shown in Table S3.

Table S3. Slopes (β) for a series of diluted samples with and without sample extraction using the kit and protocols employed for the within-assistance dog comparison in Experiment 2. The t and p values correspond to tests of the intercept against the predicted value of 0.

Peptide	Treament	β	Intercept	t	p
Oxytocin	non-extracted	.67	55.04	26.52	**< 0.01**
	extracted	1.09	1.32	.17	.87
Vasopressin	extracted	1.12	-10.67	-3.10	**.03**

For OT, the slope of the model was close to 1 when samples were extracted, and the model's intercept did not differ significantly from 0. In contrast, when samples were not extracted, the observed-expected slope was considerably below 1, and the intercept differed from 0. For AVP, following extraction the observed-expected slope was ~1, however the intercept differed marginally from 0.

Measurement of total OT and AVP

These assays employed a reduction / alkylation (R/A) and protein precipitation (PPT) procedure that we have described and validated elsewhere (Brandtzaeg et al., 2016). Briefly, this approach

irreversibly breaks disulfide bridges between peptide hormones and plasma/serum proteins, dramatically increasing detectable concentrations. Thus, while the extraction procedures described above are thought to isolate the ‘free’ hormone concentrations, measurement of total hormone concentrations may provide a better biomarker for individual differences in longer-term peptide release (Brandtzaeg et al., 2016).

Although originally developed and validated for the measurement of OT, this protocol similarly allows for the detection of much higher concentrations of AVP. Using pooled dog plasma we performed the R/A PPT procedure described above and performed a series of dilutions to assess linearity and parallelism. The expected concentration of diluted samples strongly predicted the observed concentration ($R^2 = 0.99$) however linearity and parallelism were best at the highest concentration dilutions, with non-parallel displacement against the standard curve at dilutions greater than 1:4. Therefore all samples analyzed for total AVP were measured above this threshold.

Supplemental Results

Experiment 1

Post-test free OT and AVP, and correlations with behavior during the test

Free oxytocin and vasopressin concentrations in samples collected before and after the test were highly correlated, suggesting relatively stable individual differences across the test period (OT: $R = 0.69$, $t_{40} = 6.04$, $p < 0.01$; AVP: $R = 0.67$, $t_{40} = 5.77$, $p < 0.01$).

Because the composite aggression score was strongly positively skewed (reflecting many dogs with no aggressive response) we discretized this variable by conducting a k-means cluster analysis to assign dogs to two groups representing the least and most aggressive responders. The resulting ‘high aggression’ group consisted of 9 dogs, all enrolled as cases, and the ‘low aggression’ group consisted of 33 dogs, including 13 cases and 20 controls. To assess whether behavior during the test predicted changes in free OT and AVP, we used membership in the low or high aggression groups as predictors of post-test peptide concentrations, controlling for

baseline concentrations. The extent of aggressive behavior during the test did not predict the change in free peptide levels from baseline (OT: $\chi 2$ = 0.45, df = 1, p = 0.50; AVP-Post: $\chi 2$ = 0.09, df = 1, p = 0.77), nor did either free OT or free AVP concentrations differ significantly between the two time points (paired t-tests, OT: t_{41} = 0.97, p = 0.34; AVP: t_{41} = 0.12, p = 0.90).

C-BARQ results

We used pre-test free and total OT and AVP concentrations and the pre-test free and total OT:AVP ratio as predictors of dog behavior measured by the C-BARQ. Because our hypotheses addressed links between OT, AVP and aggression, we restricted these analyses to C-BARQ factor scores for dog-directed aggression, (human) stranger-directed aggression, and dog- and human-directed fear. The latter two items were included because fear has been reported as a common motivation for aggressive behavior in dogs (Borchelt, 1983; Galac & Knol, 1997; Lindsay, 2013). For free OT and AVP, these analyses revealed a negative association between baseline free AVP and dog-directed aggression (low AVP → high aggression), with no other significant associations (Table S4). For total OT and AVP, these analyses revealed a positive association between dog-directed aggression and total AVP, with no other significant relationships (Table S4).

Table S4. Experiment 1 associations between pre-test oxytocin (OT) and vasopressin (AVP) and owner-reports of dog behavior measured with the Canine Behavioral Assessment & Research Questionnaire (C-BARQ). Significant associations are bolded.

	Free Peptide Concentration						Total Peptide Concentration					
	AVP		OT		OT:AVP Ratio		AVP		OT		OT:AVP Ratio	
	$\chi 2$	p	$\chi 2$	p	$\chi 2$	p	$\chi 2$	p	$\chi 2$	p	$\chi 2$	p
**Dog directed aggression**	5.70	**0.02**	0.05	0.82	2.29	0.13	5.20	**0.02**	0.06	0.81	2.17	0.14
**Dog directed fear**	0.41	0.52	1.24	0.26	0.77	0.38	0.24	0.63	0.37	0.55	0.49	0.48
**Stranger directed aggression**	1.27	0.26	0.01	0.91	0.11	0.74	0.31	0.58	0.00	0.98	0.17	0.68
**Stranger directed fear**	1.66	0.20	0.89	0.34	1.09	0.30	0.66	0.42	6.30	0.01	1.64	0.20

Experiment 2

CBARQ results

Within the assistance dog population there was minimal variation on all C-BARQ measures with the exception of dog-directed fear. For dog-directed aggression, human-directed aggression, and human-directed fear, all but 4 or fewer dogs received the lowest possible score, precluding statistical analyses with these measures. For dog-directed fear there were no associations with free OT or AVP (OT: $\chi 2 = 3.22$, df = 1, p = .07; AVP: $\chi 2 = 2.74$, df = 1, p = .10), but the free OT:AVP ratio was negatively associated with this measure ($\chi 2 = 5.84$, df = 1, p = 0.02).

References

Borchelt, P. L. (1983). Aggressive behavior of dogs kept as companion animals: classification and influence of sex, reproductive status and breed. *Applied Animal Ethology, 10*(1), 45-61.

Brandtzaeg, O. K., Johnsen, E., Roberg-Larsen, H., Seip, K. F., MacLean, E. L., Gesquiere, L. R., et al. (2016). Proteomics tools reveal startlingly high amounts of oxytocin in plasma and serum. *Scientific Reports*.

Cool, D. R., & DeBrosse, D. (2003). Extraction of oxytocin and arginine–vasopressin from serum and plasma for radioimmunoassay and surface-enhanced laser desorption–ionization time-of-flight mass spectrometry. *Journal of Chromatography B, 792*(2), 375-380.

Galac, S., & Knol, B. (1997). Fear-motivated aggression in dogs: patient characteristics, diagnosis and therapy. *Animal Welfare, 6*(1), 9-15.

Lindsay, S. R. (2013). *Handbook of Applied Dog Behavior and Training, Adaptation and Learning*: John Wiley & Sons.
